# Effect of Tryptophan Depletion on Conditioned Threat Memory Expression: Role of Intolerance of Uncertainty

**DOI:** 10.1016/j.bpsc.2020.12.012

**Published:** 2021-05

**Authors:** Jonathan W. Kanen, Frederique E. Arntz, Robyn Yellowlees, David M. Christmas, Annabel Price, Annemieke M. Apergis-Schoute, Barbara J. Sahakian, Rudolf N. Cardinal, Trevor W. Robbins

**Affiliations:** a Department of Psychology, University of Cambridge, Cambridge, United Kingdom; b Behavioural and Clinical Neuroscience Institute, University of Cambridge, Cambridge, United Kingdom; c Department of Psychiatry, University of Cambridge, Cambridge, United Kingdom; d Cambridgeshire and Peterborough NHS Foundation Trust, Cambridge, United Kingdom; e Section of Eating Disorders, Department of Psychological Medicine, Institute of Psychiatry, Psychology and Neuroscience, King’s College London, London, United Kingdom; f Department of Neuroscience, Psychology and Behaviour, University of Leicester, Leicester, United Kingdom; g Department of Psychology, Leiden University, Leiden, the Netherlands

**Keywords:** Emotion, Fear conditioning, Memory, Serotonin, Threat conditioning, Tryptophan depletion

## Abstract

**Background:**

Responding emotionally to danger is critical for survival. Normal functioning also requires flexible alteration of emotional responses when a threat becomes safe. Aberrant threat and safety learning occur in many psychiatric disorders, including posttraumatic stress disorder, obsessive-compulsive disorder, and schizophrenia, in which emotional responses can persist pathologically. While there is evidence that threat and safety learning can be modulated by the serotonin systems, there have been few studies in humans. We addressed a critical clinically relevant question: How does lowering serotonin affect memory retention of conditioned threat and safety memory?

**Methods:**

Forty-seven healthy participants underwent conditioning to two stimuli predictive of threat on day 1. One stimulus but not the other was subsequently presented in an extinction session. Emotional responding was assessed by the skin conductance response. On day 2, we employed acute dietary tryptophan depletion to lower serotonin temporarily, in a double-blind, placebo-controlled, randomized between-groups design. We then tested for the retention of conditioned threat and extinction memory. We also measured self-reported intolerance of uncertainty, known to modulate threat memory expression.

**Results:**

The expression of emotional memory was attenuated in participants who had undergone tryptophan depletion. Individuals who were more intolerant of uncertainty showed even greater attenuation of emotion following depletion.

**Conclusions:**

These results support the view that serotonin is involved in predicting aversive outcomes and refine our understanding of the role of serotonin in the persistence of emotional responsivity, with implications for individual differences in vulnerability to psychopathology.

Emotional responses to threats are critical for survival. Once a threat is no longer present, emotion must adapt to reflect safety for normal functioning to continue. Dysfunction of threat and safety learning lies at the core of posttraumatic stress disorder (1) and anxiety disorders (2,3) and is a feature of obsessive-compulsive disorder (4, 5, 6) and schizophrenia (7). Elucidating contributors to persistent emotional reactions is essential for developing new treatments. We tested the influence of the neuromodulator serotonin (5-HT) on the retention of conditioned threat and safety memory, with a widely used laboratory model (8).

In Pavlovian threat conditioning paradigms, more commonly known as fear conditioning (9), a neutral stimulus is paired with an aversive outcome (e.g., mild electric shock). Individuals learn that the cue signals threat, and an anticipatory sympathetic nervous system arousal response occurs. This manifests as measurable perspiration known as the skin conductance response (SCR). After learning that a cue signals threat, the stimulus can be repeatedly presented without the aversive consequence (extinction learning)—a model of exposure therapy in the clinic by which a new memory of safety should be formed. These two memories—threat and safety—compete for expression upon re-encountering a conditioned stimulus (CS) (10,11). Conditioned threat memories (learned physiological responses to conditioned stimuli) often persist despite extinction training, and re-emerge after the passage of time (spontaneous recovery) or after re-exposure to adversity (reinstatement) (10). Understanding what contributes to spontaneous recovery and reinstatement is of great clinical interest and has implications for conditions such as posttraumatic stress disorder (8,12).

Serotonin, meanwhile, is widely implicated in aversive learning (13). While several studies have begun to explore the role of serotonin in threat and safety learning and in aversive memory, most experiments have been carried out in rodents (14). The dearth of human studies at the nexus of threat memory and serotonin function is particularly surprising given that first-line pharmacological treatments of disorders in which threat conditioning processes are impaired modulate serotonin (15). No one, to our knowledge, has manipulated serotonin experimentally to examine its influence on spontaneous recovery in humans.

Acute tryptophan depletion (ATD) is commonly used to study serotonin: tryptophan, the biosynthetic precursor to serotonin, is temporarily removed from the diet in the presence of other amino acids, which decreases serotonin synthesis (16, 17, 18, 19, 20). ATD and 5-HT_2A/2C_ receptor antagonism via ritanserin have attenuated threat conditioning in humans, as assessed by SCR (21,22). 5-HT can also impact startle when anticipating shocks during acquisition (23, 24, 25). Fourteen-day administration of the serotonin reuptake inhibitor (SRI) escitalopram in humans did not impact the acquisition of threat memory but facilitated extinction (using SCR) (26). Fourteen-day treatment with fluoxetine (an SRI) in mice, initiated before extinction, diminished spontaneous recovery and reinstatement; fluoxetine was present in all postacquisition phases (27). Treatment of rats, the primary animal model, with citalopram (an SRI) for 22 days, before extinction training but after conditioning, impaired extinction, whereas 9-day treatment had no effect; spontaneous recovery and reinstatement were not studied (28). A human behavioral genetics study found a relationship between spontaneous recovery (not acquisition or extinction) and variation in the serotonin transporter polyadenylation polymorphism (29). Other human studies have shown that 5-HT modulated explicit, often same-day, memory (30, 31, 32) and processing of emotional facial expressions (33), rather than implicit memory assessed physiologically.

Another factor can influence threat conditioning processes: intolerance of uncertainty, the dispositional tendency to appraise uncertain situations as aversive (34, 35, 36, 37, 38, 39, 40, 41, 42, 43), assessed by the Intolerance of Uncertainty Scale (IUS) (44). Indeed, high IUS score has been linked to enhanced spontaneous recovery and reinstatement (34,36). Critically, effects of high intolerance of uncertainty on threat conditioning processes were distinct from trait anxiety (37,38,40, 41, 42). Intolerance of uncertainty is a transdiagnostic construct (45, 46, 47) and is heightened in individuals with diagnoses spanning generalized anxiety disorder, social phobia, panic disorder, agoraphobia, obsessive-compulsive disorder, and depression (46,47), and those with posttraumatic stress disorder symptoms (48), making the IUS score relevant for psychiatric classification frameworks (e.g., RDoC [Research Domain Criteria]) (49). To our knowledge, this study is the first to employ the IUS in the context of serotonin’s emotional effects.

Here, we employed ATD in healthy humans and investigated the following questions: How does lowering serotonin affect the retention of conditioned threat and extinction memory, and does intolerance of uncertainty influence how serotonin modulates emotion? We predicted that lowering serotonin function would modulate the expression of previously formed threat memory, without affecting expression of extinction memory, and that accounting for IUS score would contribute to explaining these effects.

## Methods and Materials

### Participants

Forty-seven healthy participants (mean age, 25 years; age range, 18–25 years; 29 males, 18 females), free from psychiatric disorder, who met criterion for Pavlovian conditioning (assessed by SCR) were included. Participants (Table 1) gave informed consent and were paid.

**Table 1:** Group Characteristics

	Placebo	Depletion
Age, Years	24.23 (5.88)	25.80 (6.24)
Education, Years	17.18 (2.38)	17.28 (2.42)
BDI-II	5.23 (4.38)	4.04 (3.92)
STAI	37.36 (7.27)	35.52 (5.96)
IUS	55.73 (13.69)	49.80 (13.44)

Values are mean (SD). The questionnaires listed here were administered before depletion took effect: BDI-II (75), STAI (55), and IUS (44).

BDI-II, Beck Depression Inventory-II; IUS, Intolerance of Uncertainty Scale; STAI, State-Trait Anxiety Inventory.

### Acute Tryptophan Depletion

Participants were randomly assigned to receive either ATD (*n =* 25; 16 males, 9 females) or placebo (*n =* 22; 13 males, 9 females) in a double-blind between-groups design. The depletion group received a drink that contained a balance of all essential amino acids except tryptophan. The placebo group received the same drink, with tryptophan (50).

### Task and Procedure

**Figure 1:**
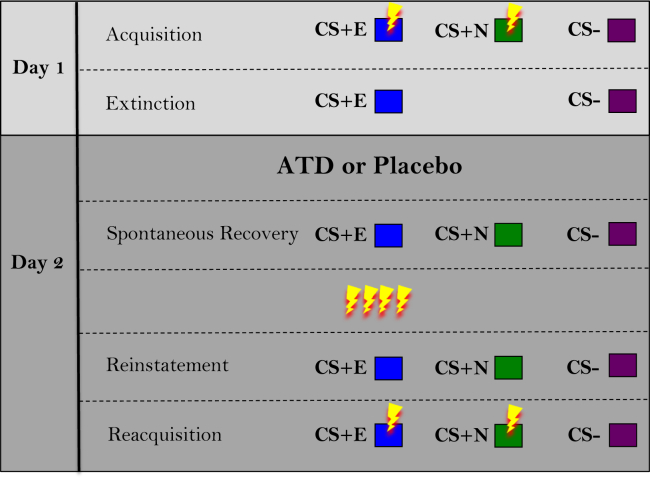
Task schematic. Each row represents a different phase of the experiment. Lightning bolts represent shock. The extinguished conditioned stimulus (CS+E) is the CS+ that was presented during the extinction phase. The not-extinguished CS+ (CS+N) is the CS+ that was not presented during the extinction phase. The CS− was never paired with shock. ATD, acute tryptophan depletion.

The protocol received ethical approval. Participants attended sessions on 2 consecutive days. Day 1 comprised a short afternoon session with no serotonergic manipulation. Participants were subjected to the threat of mild electrical stimulation (shock) (1,29,51, 52, 53), which was calibrated to be uncomfortable but not painful. Acquisition involved three conditioned stimuli (CSs): CS+E, CS+N, and CS−. Two CSs, CS+E (extinguished) and CS+N (not extinguished), were paired with receipt of shock (unconditioned stimulus [US]) on 37.5% of trials; the CS− was never paired with the US (Figure 1). Extinction followed: the CS+E and CS− were repeatedly presented, both without the US. The CS+N was not presented. On day 2, participants arrived in the morning having fasted for at least 9 hours, gave a blood sample, and ingested either the placebo or ATD drink. In the afternoon a second blood sample was taken and, at least 4.5 hours following ingestion (54), participants were re-exposed to the CS+E, CS+N, and CS− without the US, to assess spontaneous recovery to the CS+E (10). At this stage, the CS+N is a comparator against which spontaneous recovery of the CS+E can be measured. If ATD modulates expression of the original threat memory, it would be expected to alter responses to the CS+N. If it specifically affects the expression of the extinction memory (spontaneous recovery), it would be expected to alter responses to the CS+E but not to the CS+N. Reinstatement comprised four USs, not paired with any CS, followed by re-exposure to all conditioned stimuli. Reacquisition was conducted exactly as initial acquisition. Greater reacquisition can be reflective of a stronger threat memory (10). The context remained the same across both days.

## Results

### Blood and Mood

**Figure 2:**
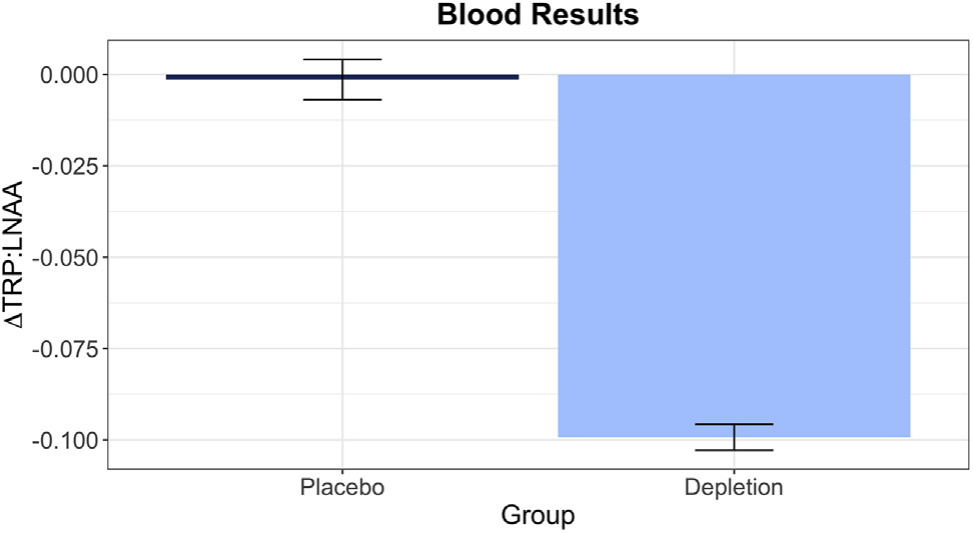
Robust tryptophan depletion was achieved, verified via plasma samples. More negative values indicate greater depletion of tryptophan. Error bars indicate ±1 SE. ΔTRP:LNAA, change in the ratio between tryptophan and all large neutral amino acids from before to after depletion.

Robust depletion of tryptophan was achieved (*t*_43_ = −15.317, *p =* 5.05 × 10^−19^) (Figure 2). Mood, assessed prior to the task, after depletion had taken effect, did not differ from those participants who received placebo (*t*_38_ = −1.227, *p = .*228).

### Acquisition Before Depletion

**Figure 3:**
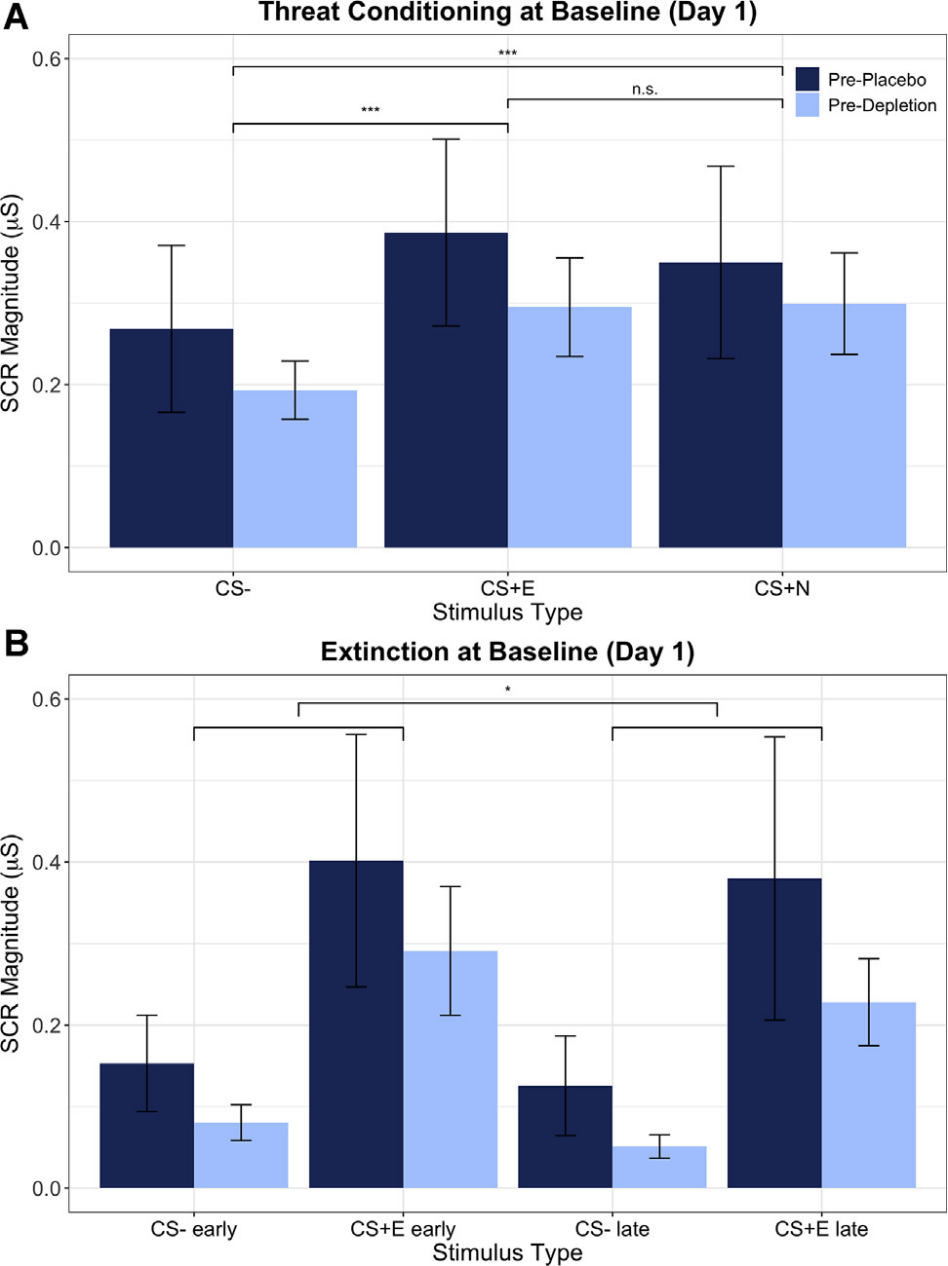
**(A)** Skin conductance responses (SCRs) in the initial threat conditioning phase (acquisition) on day 1, conducted before serotonergic challenge. There were no differences between the future placebo and future acute tryptophan depletion (ATD) groups, and both groups showed significant threat conditioning to both paired conditioned stimuli (CS+s) compared with the CS−, as predicted. This equivalent baseline conditioning on day 1 enabled testing the effects of ATD on its retention on day 2. Brackets denote follow-up *t* tests contrasting stimuli within group, after observing a main effect of stimulus. ∗∗∗*p* < .001. Error bars indicate ±1 SE. **(B)** SCRs in the extinction phase on day 1. Smaller brackets refer to the beginning and end of extinction, and the larger bracket denotes a mild reduction in SCRs in late compared with early extinction. ∗*p* < .05. Error bars indicate ±1 SE. Raw data (following transformation) (see Supplement) are displayed. CS+E, extinguished CS+; CS+N, not extinguished CS+; n.s., not significant.

Threat conditioning was achieved and was no different between those who later received placebo versus depletion (Figure 3A). Repeated-measures analysis of covariance (ANCOVA) with group assignment (future placebo, future ATD) and sex (male, female) as between-subjects factors, stimulus (CS+E, CS+N, CS−; all trials) as a within-subjects factor, and IUS as a covariate yielded a main effect of stimulus (*F*_1,56_ = 9.239, *p = .*002, η_p_^2^ = .180), no main effect of group assignment (*F*_1,42_ = 0.591, *p = .*446, η_p_^2^ = .014), and no group assignment × stimulus interaction (*F*_1,56_ = 1.272, *p = .*277, η_p_^2^ = .029). There was a significant stimulus × IUS interaction (*F*_1,56_ = 4.175, *p = .*035, η_p_^2^ = .090), no main effect of sex, and no interactions with sex (*F* < 3.5, *p* > .05, η_p_^2^ < .08). Paired *t* tests confirmed that SCRs to the CS+E (*t*_46_ = −5.315, *p =* 3 × 10^−6^) and CS+N (*t*_46_ = −4.632, *p =* 3 × 10^−5^) were each significantly greater than to the CS−.

### Extinction Before Depletion

ANCOVA was performed with group assignment (future placebo, future ATD) and sex (male, female) as between-subjects factors, stimulus (CS+E, CS−) and phase (early, late; first and last two trials) as within-subjects factors, and IUS as covariate. There was no difference during extinction between those who later received placebo versus depletion (*F*_1,42_ = 1.165, *p = .*287, η_p_^2^ = .027), nor was there an interaction between group and phase (*F*_1,42_ = 0.003, *p = .*960, η_p_^2^ = 6.10 × 10^−5^). In contrast to acquisition, there was no longer a significant effect of stimulus during the extinction session (*F*_1,42_ = 1.490, *p = .*229, η_p_^2^ = .034), indicating evidence of extinction. Additionally, there was an effect of phase (*F*_1,42_ = 4.125, *p = .*049, η_p_^2^ = .089), showing lower responding, irrespective of stimulus, in the late trials. There was no phase × stimulus interaction (*F*_1,42_ = 0.074, *p = .*787, η_p_^2^ = .002). There was no main effect of IUS, nor were there any significant interactions with IUS (*F* < 2.5, *p* > .05, η_p_^2^ < .06). There was a main effect of sex (*F*_1,42_ = 5.064, *p = .*030, η_p_^2^ = .108), such that females extinguished better than males. We therefore included sex as a factor in the core day 2 analyses.

### Spontaneous Recovery After Depletion

**Figure 4:**
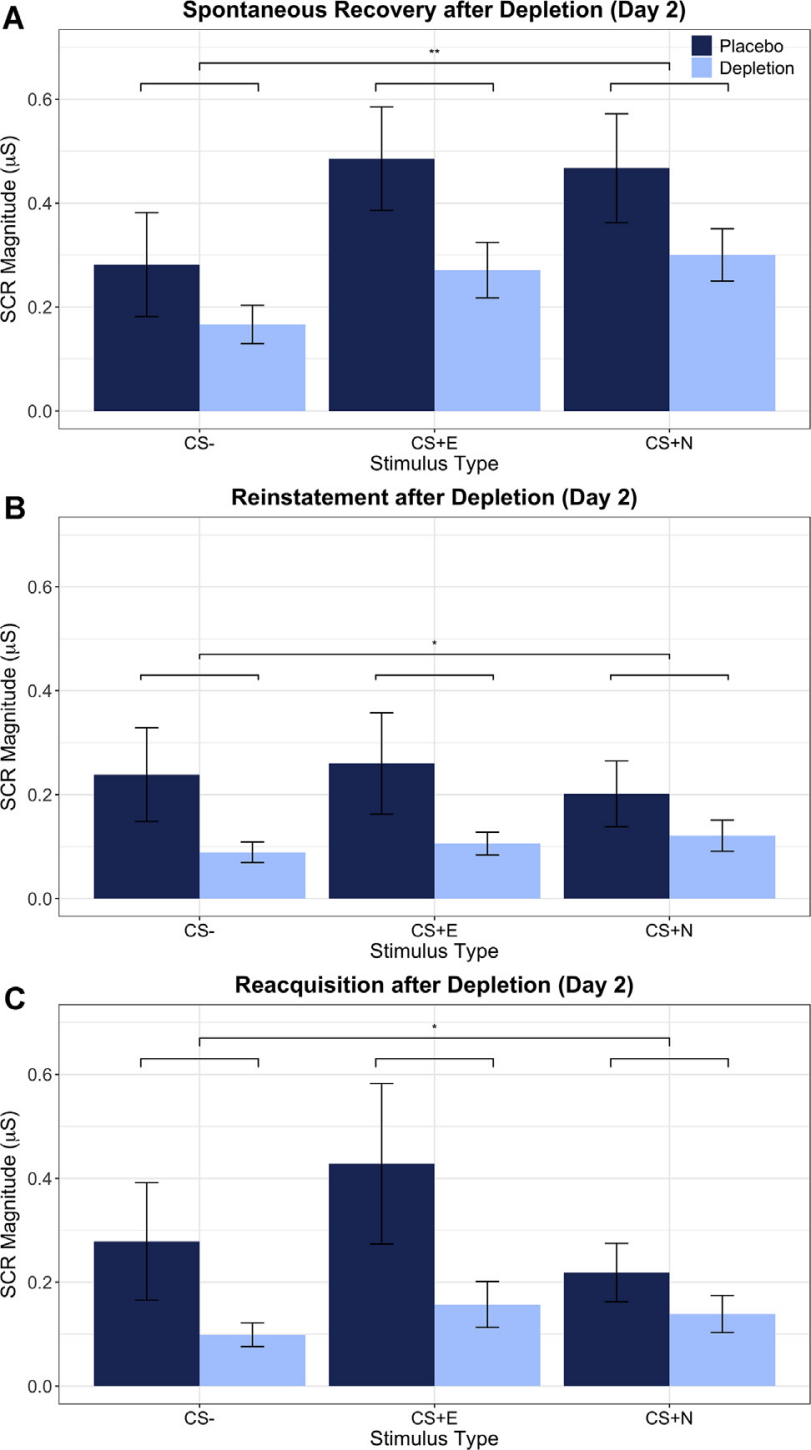
Tryptophan depletion reduced skin conductance response (SCR) expression. SCRs are displayed from the **(A)** spontaneous recovery, **(B)** reinstatement, and **(C)** reacquisition phases. Large brackets denote a main effect of stimulus. ∗∗*p* < .01; ∗*p* < .05. Error bars indicate ±1 SE. Raw data (following transformation) (see Supplement) are displayed, not adjusted values after controlling for intolerance of uncertainty, sex, or strength of initial conditioning on day 1. CS, conditioned stimulus; CS+E, extinguished CS+; CS+N, not extinguished CS+.

ATD modulated emotional responses during the spontaneous recovery phase (Figure 4A). ANCOVA was conducted with serotonin status (placebo, ATD), sex (male, female), and stimulus (CS+E, CS+N, CS−; first half of trials) as factors, controlling for strength of initial conditioning and IUS. SCR during acquisition was used as a covariate because we were interested in assessing the influence of ATD on memory expression, irrespective of how the strength of the initial memory affected expression a day later. IUS was used as an additional covariate because this trait can affect threat memory expression (34). There was a significant main effect of serotonin status (*F*_1,41_ = 7.729, *p = .*008, η_p_^2^ = .159)—emotional responses were significantly attenuated under ATD. There was a main effect of stimulus (*F*_2,68_ = 3.750, *p = .*036, η_p_^2^ = .084). There was no main effect of sex, nor were there any significant interactions with sex (*F* < 1.1, *p* > .05, η_p_^2^ < .03). The strength of acquisition covariate was significant (*F*_1,41_ = 140.487, *p =* 1.311 × 10^−16^, η_p_^2^ = .815). Rerunning the ANCOVA without IUS as a covariate also yielded a significant main effect of serotonin status (*F*_1,42_ = 5.406, *p = .*025, η_p_^2^ = .114). IUS, furthermore, was a significant predictor over and above trait anxiety (55) (see Supplement). Paired *t* tests revealed that responses to the CS+E and CS+N, collapsed across serotonergic status, were each significantly greater than responses to the CS− (CS+E [*t*_46_ = −4.549, *p =* 3.9 × 10^−5^], CS+N [*t*_46_ = −5.089, *p =* 7 × 10^−6^]), demonstrating that return of threat memory expression occurred irrespective of serotonin status. Responses to the CS+E and CS+N did not differ from one another (*t*_46_ = −0.312, *p = .*756), likely because there was not robust extinction of the CS+E. There was no serotonin × stimulus interaction (*F*_2,68_ = 1.916, *p = .*162, η_p_^2^ = .045), indicating that the effect of ATD was not specific to any CS. Conditioning to both CS+s from day 1 was retained on day 2 in both the placebo and ATD groups; however, overall emotional responsivity was diminished by ATD, irrespective of stimulus.

### Relationship Between Spontaneous Recovery and Extent of Depletion

**Figure 5:**
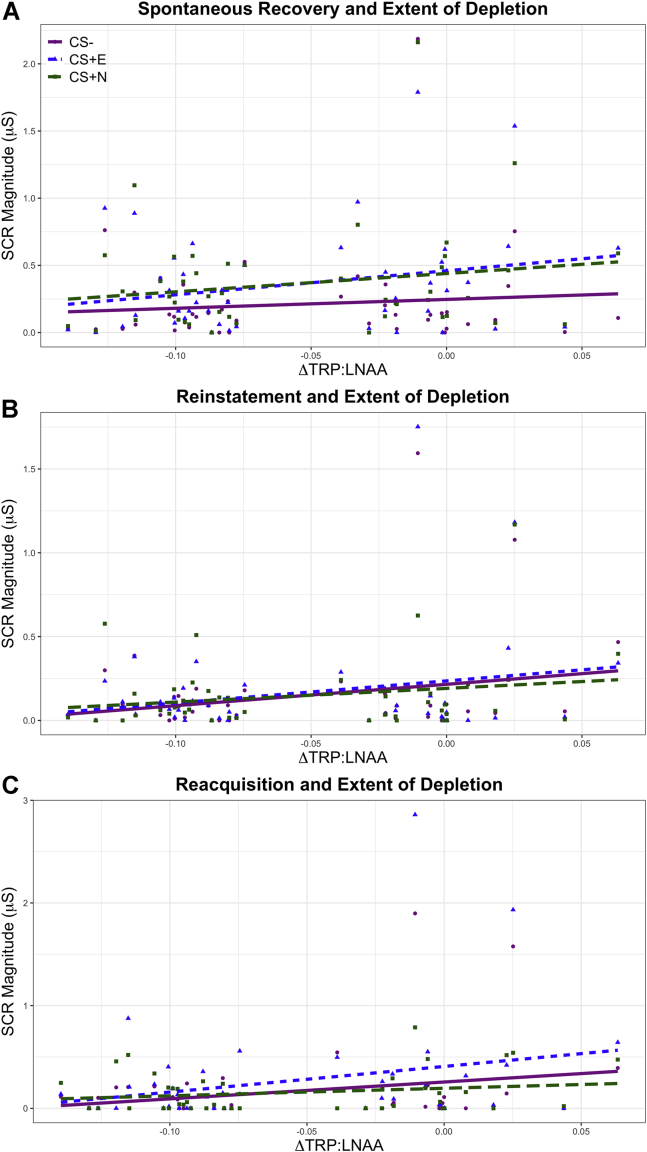
Skin conductance responses (SCRs) on day 2 plotted against the extent of depletion during the **(A)** spontaneous recovery, **(B)** reinstatement, and **(C)** reacquisition phases. Lower x-axis values indicate greater depletion, indexed by the change in the ratio of tryptophan to large neutral amino acids (ΔTRP:LNAA) assessed via plasma samples. Raw data (following transformation) (see Supplement) are displayed, not adjusted values after controlling for intolerance of uncertainty, sex, or strength of initial conditioning on day 1. The CS− is denoted by purple circles, the extinguished CS+ (CS+E) is denoted by blue triangles, and the not-extinguished CS+ (CS+N) is denoted by green squares. Significant relationships between depletion and SCR were seen in the spontaneous recovery phase for the CS+E and CS+N but not for the CS−, in reinstatement for the CS+E and CS−, and in reacquisition for the CS+E and CS−. CS, conditioned stimulus.

The extent of tryptophan depletion significantly correlated with the attenuation of threat responding but not with safety memory expression during the spontaneous recovery phase (Figure 5A). Critically, this substantiated the relationship between depletion and conditioned threat memory expression during spontaneous recovery. Using a partial correlation to control for strength of acquisition, IUS, and sex, there was a significant relationship between the degree of tryptophan depletion overall (including both placebo and ATD conditions) and the extent to which the threat memory returned. The extent of depletion correlated with the SCR to the CS+E and CS+N, and not to the CS−, indicating that the effect of tryptophan depletion did not generalize to safety memory expression: threat memory responses were more attenuated the greater the depletion (CS+E [*r*_40_ = .413, *p = .*007], CS+N [*r*_40_ = .389, *p = .*011], CS− [*r*_40_ = .113, *p = .*478]), which remained significant after being subjected to the Benjamini–Hochberg procedure at *q* = .15 for three comparisons (56). These relationships did not reach significance in the ATD condition alone (*p* > .05). However, there was no interaction between stimulus (CS+E, CS+N, CS−) and plasma results on SCR, as assessed by ANCOVA with plasma values, IUS score, sex, and strength of initial conditioning as predictors (*F*_2,67_ = 2.315, *p = .*115, η_p_^2^ = .055).

### Role of Intolerance of Uncertainty in ATD Effects on Spontaneous Recovery

**Figure 6:**
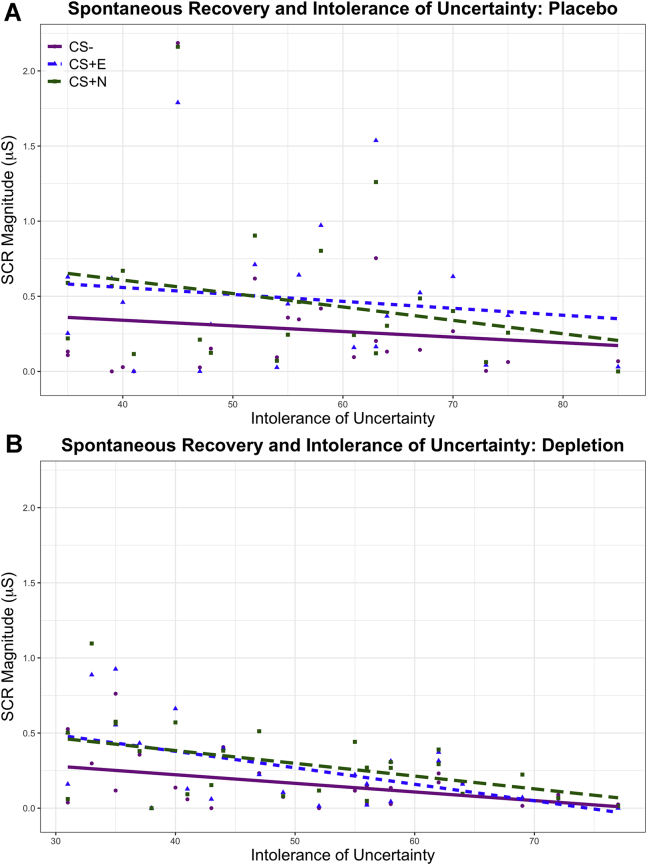
Skin conductance responses (SCRs) during spontaneous recovery (day 2) plotted against self-report on the Intolerance of Uncertainty Scale (IUS), shown separately for **(A)** placebo and **(B)** depletion. Raw data (following transformation) (see Supplement) are displayed. The CS− is denoted by purple circles, the extinguished CS+ (CS+E) is denoted by blue triangles, and the not-extinguished CS+ (CS+N) is denoted by green squares. CS, conditioned stimulus.

Next, we examined how IUS score related to SCR during spontaneous recovery (Figure 6A). Correlation analyses between IUS score and SCR to each CS, controlling for strength of conditioning, revealed significant effects in the depletion group only: individuals more intolerant of uncertainty showed significantly diminished emotional expression to the CS+E (*r*_25_ = −.554, *p = .*004), CS+N (*r*_25_ = −.453, *p = .*023), and CS− (*r*_25_ = −.418, *p = .*038). Under placebo, this relationship with IUS score was not present (CS+E [*r*_25_ = −.135, *p = .*549], CS+N [*r*_25_ = −.249, *p = .*264], CS− [*r*_25_ = −.109, *p = .*629]). Critically, these results survived correction for six comparisons (56). Next, an interaction term between serotonin and IUS score was incorporated into the general linear model used in the initial analysis of spontaneous recovery, to examine whether IUS score and serotonin status interacted to modulate SCR to specific stimuli. ANCOVA with serotonin and IUS score as a between-subjects interaction term, controlling for main effects and strength of initial conditioning, sex (male, female) as an additional between-subjects factor, and stimulus (CS+E, CS+N, CS−) as within-subjects factors, did not show an interaction between serotonin and IUS score (*F*_1,41_ = 0.058, *p = .*811, η_p_^2^ = .001) or between serotonin, IUS score, and stimulus (*F*_2,67_ = 1.278, *p = .*281, η_p_^2^ = .030). While there was no interaction between ATD and IUS score, the correlation results suggest that ATD modulated the relationship between IUS score and SCR to the conditioned stimuli.

### Reinstatement After Depletion

The SCRs to the US during reinstatement were unaffected by ATD (*F*_1,39_ = 0.729, *p = .*399, η_p_^2^ = .018) (Figure 4B). Instead, ATD modulated cue-evoked SCRs during the reinstatement phase of the experiment. ANCOVA revealed a significant effect of serotonin status (*F*_1,39_ = 4.403, *p = .*042, η_p_^2^ = .101), again with lower SCRs under ATD. By this stage in the experiment, however, there was no longer a main effect of stimulus (*F*_1,47_ = 0.083, *p = .*823, η_p_^2^ = .002); there was no differential response to the CS+s relative to the CS−, and thus no reinstatement of the threat memory. There was also no serotonin × stimulus interaction (*F*_1,47_ = 1.306, *p = .*267, η_p_^2^ = .032).

### Relationship Between Reinstatement and Extent of Depletion

Controlling for strength of acquisition, IUS score, and sex, there was a significant correlation between depletion and CS+E and CS− responses but not between depletion and CS+N responses (CS+E [*r*_38_ = .413, *p = .*008], CS+N [*r*_38_ = .234, *p = .*146], and CS− [*r*_38_ = .455, *p = .*003]) (Figure 5B). These results were not predicted and need further investigation in future studies.

### Reacquisition After Depletion

Because effects in these paradigms are often short lived (57), and participants’ SCRs tended to habituate later in the experiment, the same analysis used in the spontaneous recovery and reinstatement phases was repeated on the first two trials of the reacquisition phase, which showed a main effect of serotonin status: responses to the conditioned stimuli were attenuated overall by ATD (*F*_1,39_ = 6.974, *p = .*012, η_p_^2^ = .152) (Figure 4C). There was no main effect of stimulus (*F*_2,78_ = 1.598, *p = .*209, η_p_^2^ = .039), nor was there a serotonin × stimulus interaction (*F*_2,78_ = 2.194, *p = .*118, η_p_^2^ = .053), providing no evidence of reconditioning in either group.

### Relationship Between Reacquisition and Extent of Depletion

A partial correlation analysis was performed, as in the prior phases (accounting for sex), isolating the first two trials, as before. There was a significant correlation between depletion and SCRs to the CS+E and CS−, but not to the CS+N (CS+E [*r*_38_ = .475, *p = .*002], CS+N [*r*_38_ = .229, *p = .*156], CS− [*r*_38_ = .371, *p = .*018]) (Figure 5C). These results were not predicted and require further investigation in future studies.

### Summary of Results

Baseline conditioning and extinction did not differ between those destined to receive placebo versus ATD. The key result was that ATD attenuated the expression of previously acquired emotion in the spontaneous recovery phase. While the reduction in SCR during the spontaneous recovery phase by ATD was not specific to any of the three stimuli at the group level, the greater the extent of depletion, the more the CS+E and CS+N were attenuated, whereas there was no such correlation for SCRs to the CS−. Differential conditioning was not abolished by ATD, and accounting for IUS score contributed to the prediction of how ATD modulated SCR during spontaneous recovery. Following ATD, individuals more intolerant of uncertainty showed significantly less emotional expression to all three stimuli during the spontaneous recovery phase. Importantly, SCR to the US was unaffected by ATD. ATD also attenuated responses during the reinstatement and reacquisition phases, consistent with the spontaneous recovery phase results; however, there was no longer evidence of differential conditioning.

## Discussion

Here we showed, for the first time, that modulating serotonin affected the expression of aversive emotional memory in humans. During the key phase of the study—spontaneous recovery—ATD diminished physiological responses to the CS+s and CS− nonspecifically, and differential conditioning was preserved. Analysis of individual plasma samples, however, revealed that a greater degree of depletion was associated with reduced emotional responding to the CS+s, with no effect on CS− responses. These plasma data suggest that aversive emotional memory was attenuated by ATD. Examining intolerance of uncertainty, a trait previously related to spontaneous recovery (34), aided in uncovering how ATD affected emotion by contributing to the prediction of the general linear model. Individuals with a higher IUS score showed even lower responses during spontaneous recovery when depleted. ATD also attenuated cue-evoked SCRs during the reinstatement and reacquisition phases. Importantly, unconditioned responses were unaffected by ATD, indicating that the effect was specific to learned cues and not a general blunting of arousal encompassing responses to aversion itself. Mood was unaffected by ATD, consistent with previous studies of healthy volunteers (58, 59, 60). By using a task that elicited physiological reactions, however, it was possible to uncover an effect of serotonin on emotion. The primary implication of the study is that serotonin plays a central role in conditioned threat memory expression. Excessive serotonin signaling may be an important contributor to the persistence of pathological emotional reactions. This might be a feature of individuals who are highly intolerant of uncertainty, a trait we propose could represent a latent marker of vulnerability to serotonergic dysregulation.

The directionality of the depletion effects—a reduction, rather than enhancement, of emotion—may seem counterintuitive. These results, however, are in line with and advance influential theories of serotonin function (61, 62, 63) and are consistent with an array of experimental data (14,21,22,62,64). Serotonin is thought to be critically involved in predicting punishment, and aversively conditioned cues stimulate serotonin release (14,62, 63, 64). The present results are most directly comparable to, and therefore substantiated by, two studies that diminished serotonin function in healthy humans and showed attenuated SCR during threat conditioning of neutral cues (21,22). One of these studies additionally employed functional magnetic resonance imaging and found that the attenuation of SCR following ATD was accompanied by diminished signals in the amygdala and orbitofrontal cortex that were otherwise evoked by cues predictive of aversion (21). The current study represents an important extension of this work on initial conditioning in humans by addressing a critical clinically relevant question: How does lowering serotonin impact the intensity with which previously formed emotional memories return? Our results are also consistent with a study that conditioned rats off-drug and tested them a day later for threat memory expression under a different serotonergic manipulation. Acute SRI administration, which increases extracellular serotonin, enhanced conditioned threat memory expression, and this effect was blocked by administering a 5-HT_2C_ antagonist (but not by a 5-HT_3_ antagonist) (65). Indeed, downregulation of 5-HT_2C_ receptors is believed to occur with repeated administration of SRIs and may contribute to their therapeutic effects (62).

The present results appear to agree with what is known about the basic serotonergic innervation of different amygdala subnuclei. The basolateral nucleus of the amygdala (BLA) is critical for storing associations between cues and aversive outcomes (66). The central nucleus of the amygdala (CeA), meanwhile, is a major source of Pavlovian conditioned output from the amygdala and signals downstream to structures, including the hypothalamus and periacqueductal gray, that contribute to defensive reactions such as perspiration in humans and freezing in rodents (66). Critically, the BLA receives dense serotonergic innervation, while the CeA receives weak serotonergic input (14). Indeed, overexpressing 5-HT_2C_ receptors in the BLA and infusing a 5-HT_2A/2C_ agonist into the BLA enhanced defensive behaviors in rodents, whereas 5-HT_2C_ knockout mice displayed the opposite behavioral effect (14). This is remarkably consistent with the present findings: emotional responses to predictive cues (conditioned stimuli), which should heavily engage serotonin signaling in the BLA, were modulated by ATD, whereas SCR to the aversive outcome itself was unaffected. Indeed, it is the CeA that responds to aversive outcomes (67). That activity associated with aversive expectations occurs in the BLA, but not in the CeA, has furthermore been associated with individual differences in trait anxiety in humans (67). Meanwhile, it should be noted that in the absence of stimuli, serotonin has an inhibitory role in the lateral amygdala of rats (68,69): this accords with the view that the amygdala is a relatively “silent” brain structure, containing a strong inhibitory network to minimize firing of cells spontaneously or to irrelevant stimuli (70).

Group-level SCR during the spontaneous recovery phase was lower under ATD for the CS−, as well as for the CS+s. While the CS− is safe and thus typically associated with lower SCRs regardless of ATD, the CS− can still evoke SCRs: anticipatory arousal may be diminished but not entirely absent. The implication, based on our data, is that normally occurring nonzero CS− responses (anticipatory arousal) are serotonergically mediated and thus attenuated by ATD. In the reinstatement and reacquisition phases, in which there was no longer a differential response to the CS+s relative to the CS− regardless of serotonin status, likely owing to the short-lived nature of effects in these paradigms (57), a similar logic applies: the nonspecific attenuation of cue-evoked SCRs by ATD appears to reflect serotonin-mediated anticipatory arousal. In other words, group-level analyses of the spontaneous recovery, reinstatement, and reacquisition phases show that ATD attenuated anticipatory arousal elicited by both the CS+ and CS−.

An important limitation is that we did not see robust evidence for complete extinction on day 1. One reason could be the use of partial reinforcement during acquisition, which can prolong conditioning (43,71,72). We employed two CS+s to compare retention of conditioning versus retention of extinction: ultimately, these could not be definitively parsed. While the lack of difference between the CS+E and CS+N on day 2 is likely due to incomplete extinction, it is also possible (10) that including the CS+N in all day 2 phases cued memory for conditioning on day 1—thus enhancing memory expression for CS+E—more so than an extinction memory trace. SCR habituated during reinstatement and reacquisition, which often occurs (57), but made it more difficult to ascertain effects. The ATD group tended to have nonsignificantly lower SCRs even before depletion: while this could possibly have affected results after depletion, a covariate was included to control for this possibility. The distribution of SCR values, furthermore, was highly variable, even after appropriate transformation.

Another limitation is that serotonin was not measured directly. ATD as a method has been critiqued (73), yet defended on the basis of considerable evidence (18,20). Consonant results from human ATD studies and rodent experiments with 5,7-dihydroxytryptamine (50,74), which induces profound serotonin loss, bolsters the case that ATD reduces central serotonin.

We have shown for the first time that lowering serotonin attenuated the subsequent return of threat responses, conditioned prior to depletion: this has particular clinical relevance and advances the human literature on serotonin and threat conditioning (5,7,8,12,14). Integrating traits and neurochemical state is relevant for understanding vulnerability in health and may inform transdiagnostic mechanisms of illness to refine psychiatric classification (49) and help direct treatment strategies.
