# Supplementary material for: Effect of Tryptophan Depletion on Conditioned Threat Memory Expression: Role of Intolerance of Uncertainty
Source: Biol Psychiatry Cogn Neurosci Neuroimaging. 2021 May;6(5):590–8. doi: 10.1016/j.bpsc.2020.12.012 (PMC8099731; doi:10.1016/j.bpsc.2020.12.012)
Supplement: Supplementary Materials [file mmc1.pdf]

Effect of Tryptophan Depletion on Conditioned Threat Memory Expression: Role of Intolerance of Uncertainty

Supplemental Information

Inclusion criteria

Conditioning data from Day 1 were evaluated to determine whether participants qualified to continue with Day 2 of the conditioning experiment. Participants were said to have conditioned if they showed greater responses to both CS+s than to the CS-, averaged across either the entire acquisition phase, the first half of acquisition or the latter half of acquisition.

Exclusion criteria

Individuals who reported, during screening, having a first-degree relative (parent or sibling) with a psychiatric disorder were also excluded. Participants reported not taking any regular medication (aside from contraceptive pills), had never taken psychiatric or neurological medications, and did not have any neurological conditions. Psychiatric screening was conducted using the Mini-International Neuropsychiatric Interview (MINI) (1). Other exclusion criteria included pregnancy, past use of endocrine medication, use of St John's Wort, regular consumption of over 38 units of alcohol per week, consumption of more than five cigarettes per day, use of cannabis more than once per month, use of other recreational drugs besides cannabis more than five times in the lifespan, cardiac or circulation problems, respiratory issues including asthma, gastrointestinal disorders, kidney disorders, thyroid problems, head injury, a bleeding disorder, and diabetes.

Skin conductance

Electrodes were attached to the distal phalanges of the index and middle finger on the other arm to the shock electrode, which was counterbalanced. Base to peak increase in SCR to the CSs was valid if an increase began within a window of .5 to 4.5 seconds after stimulus onset. There was not a minimum or maximum duration of time for a response to be valid, so long as the increase plateaued before the subsequent trial. Amplitude was measured in

microsiemens (μS). SCR to the CSs and US were low-pass filtered, smoothed, and square root transformed to normalize the distribution. SCR to CSs across all phases were divided by the average SCR to the US on Day 1 to enable between-subjects comparisons (2).

Procedure

The protocol was approved by the Cambridge Central Research Ethics Committee (UK National Health Service Ethics reference 16/EE/0101). The study took place at the National Institute for Health Research / Wellcome Trust Clinical Research Facility at Addenbrooke's Hospital in Cambridge, England. Participants were paid £12.50/hour for their participation

On Day 1, participants completed baseline questionnaires, two other unrelated non-emotional computer tasks not reported here, followed by the first part of the Pavlovian task. Two non-emotional tasks were administered on Day 1: one assessed working memory and the other tested motor skills. The questionnaires administered were the Positive and Negative Affect Schedule (PANAS) (3), National Adult Reading Test (NART) (4), and a 16-item visual analogue scale (VAS). The VAS was administered to assess mood and other feelings including alertness, at the beginning of Day 1, and at the beginning, middle, and end of the main testing session on Day 2. Participants completed the Pavlovian task on Day 2 along with multiple other tasks that will be reported elsewhere.

Additional threat conditioning task details

Shock was administered to the medial wrist. During acquisition, two coloured square images (2 conditioned stimuli [CSs], denoted CS+E and CS+N) were presented for 4 seconds, on 16 trials each, and paired with receipt of shock (unconditioned stimulus; US) on 37.5% (6 of 16) of the presentations, while another coloured square image (CS-) was presented for 10 trials and was never paired with the US. After a few trials of this procedure it is normal for participants to show an anticipatory arousal response (reflected by mild perspiration of the fingers and measured by the SCR) upon viewing the CS+s, relative to the CS-. SCR measurement allowed verification that Pavlovian threat learning had occurred.

The CS+E and CS- were presented during extinction for 10 trials each. During the spontaneous recovery and reinstatement phases, the CS+E, CS+N, and CS- were presented for 10 trials each. In reacquisition, the CS+E and CS+N were once again paired with the US on 37.5% of trials (the CS- was also presented, without shock, as before). The number of trials and reinforcement schedule during the reacquisition phase were exactly as in the initial acquisition phase on Day 1.

The task design was adapted from previous threat conditioning studies (2,5–8). For the CS+ stimuli, the US occurred 3800 milliseconds after stimulus onset, and lasted 200 milliseconds, co-terminating with the image. There was an inter-trial interval (ITI) that averaged 10 seconds. The squares were colored blue, green, and purple, were counter-balanced, and were presented in a pseudo-randomized order. There was no break between acquisition and extinction, and these two phases were only separated by an ITI. There were no breaks between phases on Day 2: each phase was separated only by an ITI.

Statistics

Data were analysed using MATLAB (MathWorks) and SPSS (IBM). The Greenhouse-Geisser correction was used where applicable, in designs with within-subjects factors, to correct for violation of the sphericity assumption as determined by Mauchly's test.

Additional participant information

The number of males and females did not differ between the placebo and depletion groups (Fisher's exact test, $p = .771$).

Intolerance of Uncertainty Scale

In the IUS, individuals are presented with a series of statements describing how people might react to life's uncertainties and are asked to rate the extent to which each item is representative of them (9).

Additional rating data

Mood rating data, reported in the main text, was collected from 40 participants (n = 21 depletion), who rated how happy or sad they were feeling prior to the task, after depletion, using a VAS.

We collected rating data from 44 participants (n = 25 on depletion) on how much they enjoyed the task each day. Using a repeated measures ANOVA with serotonin status (placebo, ATD) and day (day 1, day 2) as factors, there was no main effect of ATD ($F_{(1,42)} = 1.339$, $p = .254$) nor was there a serotonin × day interaction ($F_{(1,42)} = 0.492$, $p = .487$). We also collected rating data from 37 participants (n = 20 on depletion) asking how uncomfortable they found the shocks on each day. Repeated measures ANOVA with group assignment (placebo versus ATD) and day (day 1, day 2) as factors showed there was no main effect of ATD ($F_{(1,35)} = 0.148$, $p = .703$) nor was there a group × day interaction ($F_{(1,35)} = 0.339$, $p = .564$).

Amino acids and plasma analysis

Quantities of the amino acids administered were based on previous work (10). Plasma samples were analysed using high performance liquid chromatography (HPLC). The ratio of tryptophan to large neutral amino acids (TRP:LNAAs; valine, methionine, isoleucine, leucine, tyrosine, and phenylalanine) was calculated, as this is thought to be most reflective of the extent of brain 5-HT depletion (11). A t-test was performed on the change in the TRP:LNAA ratio between samples taken at baseline and approximately 4.5 hours following administration of the mixture. Plasma levels were unavailable for two participants: one due to a staff processing error, and one due to unsuccessful venepuncture.

Correlations between IUS and acquisition

Follow-up correlation analyses, collapsed across group assignment, indicated that those who were higher in intolerance of uncertainty displayed worse cue discrimination for the CS+E (correlation between IUS and SCR to the CS+E minus CS- $r_{(47)} = -.327$, $p = .025$) whereas there was no correlation between IUS and SCR to the CS+N minus CS- ($r_{(47)}$ =

-.197, p = .184). The relationship between IUS and SCR during acquisition is depicted in Supplementary Figure 1, separated by group assignment.

Contribution of IUS and STAI to acquisition: multiple regression

Multiple regression was performed to determine the contribution of the IUS and STAI to SCR during acquisition on Day 1. IUS and STAI were the only two predictors assessed. Neither IUS nor STAI predicted differential conditioning to the CS+N (CS+N minus CS-; standardized regression coefficient β = -.217, p = .218 for IUS; β = .038, p = .829 for STAI). IUS was predictive of differential conditioning to the CS+E (CS+E minus CS-; β = -.397, p = .022 for IUS; β = .132, p = .432 for STAI). When analysing each stimulus separately, neither IUS nor STAI was predictive of SCR during acquisition to the CS+N (β = -.053, p = .761 for IUS; β = -.176, p = .316 for STAI), CS+E (β = -.114, p = .511 for IUS; β = -.149, p = .393 for STAI), or CS- (β = .021, p = .906 for IUS; β = -.234, p = .183 for STAI).

Extinction

Follow-up tests indicated the main effect of sex was driven by significantly lower SCR in females during late extinction ($F_{(1,45)}$ = 4.650, p = .036, η_p^2 = .094). There were no interactions with the factor sex during extinction (F < 3.6, p > .05, η_p^2 < .08, for all terms involving sex).

Role of intolerance of uncertainty and sex in the effects of ATD on spontaneous recovery

There was no main effect of sex nor were there any significant interactions with sex (F < 1.0, p > .05, η_p^2 < .03, for all terms involving sex) for the ANCOVA testing an interaction between IUS and serotonin on spontaneous recovery.

Relationship between effects of intolerance of uncertainty and trait anxiety: ANCOVA

IUS was highly correlated with trait anxiety (STAI) ($r_{(47)}$ = .529, p = 1.32×10^{-4}). An additional ANCOVA was therefore conducted, with serotonin status (placebo, depletion)

and sex (male, female) as between-subjects factors, stimulus (CS+E, CS+N, CS-) as within-subjects factors, with STAI, IUS, and strength of conditioning on Day 1 as covariates. Whilst including STAI as an additional covariate in an ANCOVA assessing spontaneous recovery (the primary phase of interest in this study) reproduced the key main effect of serotonin status ($F_{(1,40)} = 7.224$, $p = .010$, $\eta_p^2 = .153$), STAI was not a significant predictor over and above IUS ($F_{(1,40)} = .756$, $p = .390$, $\eta_p^2 = .019$). IUS, on the other hand, was a significant predictor over and above STAI ($F_{(1,40)} = 4.201$, $p = .047$, $\eta_p^2 = .095$). When incorporating STAI into the model, moreover, there was no longer a simple main effect of stimulus ($F_{(2,67)} = 2.771$, $p = .079$, $\eta_p^2 = .065$).

Contribution of IUS and STAI to spontaneous recovery: multiple regression

Multiple regression was performed to determine the contribution of the IUS and STAI to SCR during spontaneous recovery, collapsed across serotonin status. Aside from IUS and STAI, the additional predictors were serotonin status and strength of conditioning on Day 1. This multiple regression was conducted three times, once per stimulus type. The contribution of IUS to the prediction of SCR to the CS+N was significant (standardized regression coefficient $\beta = -.182$, $p = .013$) whereas STAI was not a significant predictor ($\beta = .06$, $p = .403$). The pattern was similar for predicting SCR to the CS+E ($\beta = -.15$, $p = .097$ for IUS; $\beta = .022$, $p = 807$ for STAI). Neither IUS nor STAI predicted SCR to the CS- ($\beta = -.09$, $p = .378$ for IUS; $\beta = .097$, $p = .342$). IUS and STAI were not predictive of SCR difference scores during spontaneous recovery for the contrast CS+N minus CS- ($\beta = -.185$, $p = .290$ for IUS; $\beta = -.050$, $p = .771$ for STAI) or CS+E minus CS- ($\beta = -.115$, $p = .514$ for IUS; $\beta = -.112$, $p = .526$ for STAI).

Effects of tryptophan depletion on unconditioned responses

The unconditioned response (UR) to the shock (US) was analysed to evaluate whether ATD reduced emotional responsiveness in general, or whether the attenuation was specific to cue-evoked emotion. To do this, SCR to the four US presentations during the reinstatement procedure (where shocks were presented as discrete events, unpaired to any CS) were isolated and averaged. Two participants were not included in reinstatement and reacquisition analyses: one due to an equipment failure, one due to experimenter error.

Univariate ANCOVA with serotonin status (placebo, depletion) and sex (male, female) as between-subjects factors, controlling for the magnitude of URs on Day 1 (at baseline) and IUS was conducted to show that responses to the unconditioned stimulus, presented as part of the reinstatement test, were not modulated by ATD. There was no interaction between serotonin status and sex ($F_{(1,39)} = .493$, $p = .487$, $\eta_p^2 = .012$). URs on Day 2, moreover, were not correlated with the extent of depletion (bivariate correlation, $r_{(43)} = .197$, $p = .205$), which was also the case when controlling for magnitude of URs on Day 1, intolerance of uncertainty, and sex (partial correlation, $r_{(38)} = .303$, $p = .058$).

Effects of tryptophan depletion on reinstatement

The reinstatement phase was analysed in the same way as the spontaneous recovery phase, examining the first half of trials, and controlling for strength of acquisition, IUS, and sex. There was no main effect of sex nor were there any significant interactions with sex ($F < .7$, $p > .05$, $\eta_p^2 < .02$, for all terms involving sex). Rerunning the ANCOVA without the IUS as a covariate also yielded a significant main effect of serotonin status ($F_{(1,40)} = 5.008$, $p = .031$, $\eta_p^2 = .111$).

Effects of tryptophan depletion on reacquisition

For the final phase of the experiment, the same analysis used in the spontaneous recovery and reinstatement phases was repeated, which examined the first half of trials. Repeated measures ANCOVA showed a non-significant effect of serotonin status ($F_{(1,39)} = 3.459$, $p = .07$, $\eta_p^2 = .081$). There was no main effect of sex nor were there any significant interactions with sex ($F < .14$, $p > .05$, $\eta_p^2 < .004$, for all terms involving sex). There was no main effect of stimulus ($F_{(1,57)} = 1.800$, $p = .182$, $\eta_p^2 = .044$) nor a serotonin × stimulus interaction ($F_{(1,57)} = .107$, $p = .\ 838$, $\eta_p^2 = .003$). Therefore, there was no evidence of re-conditioning in either the placebo or depletion groups. In both groups, however, the CS+E was numerically greater than the CS-, while the CS+N and CS- were numerically similar (in both the analysis of the first half of trials and the first two trials). For the ANCOVA performed on the first two trials, reported in the main text, there was no main effect of sex nor were there any significant interactions with sex ($F < .4$, $p > .05$, $\eta_p^2 < .01$, for all terms involving sex).

Relationship between reacquisition and extent of tryptophan depletion

Focusing on the first half of trials in the reacquisition phase, there were significant correlations between the extent of depletion and responses to each of the three stimuli (CS+E, $r_{(38)} = .340$, $p = .032$; CS+N, $r_{(38)} = .356$, $p = .024$; CS-, $r_{(38)} = .336$, $p = .034$).

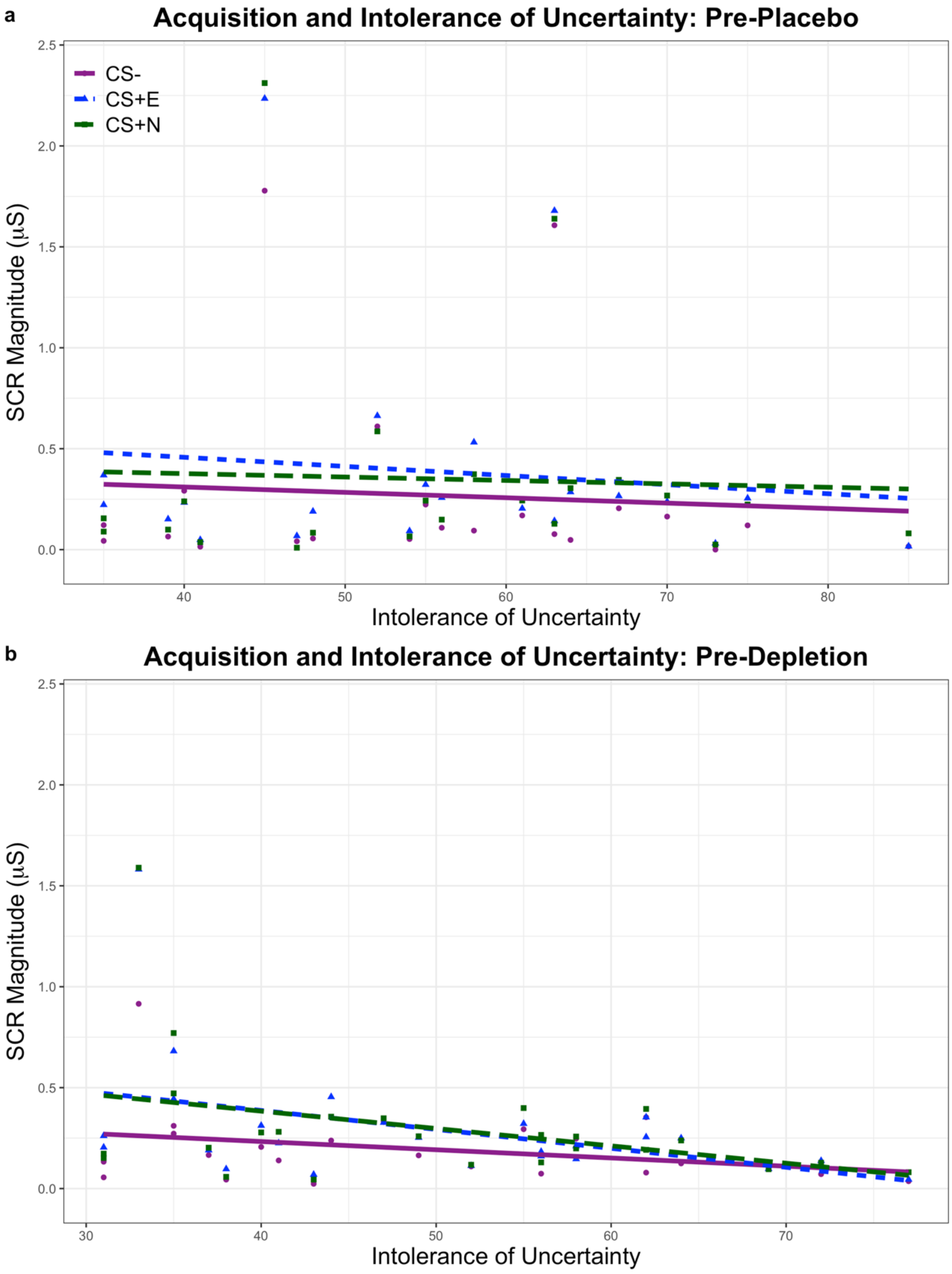

Figure S1. Skin conductance responses (SCR) during acquisition (Day 1) plotted against self-report on the intolerance of uncertainty scale (IUS), shown separately for the groups destined to receive **(A)** placebo and **(B)** depletion. Raw data (following transformation) are displayed. µS = microsiemens. CS- denoted by purple circles, CS+E (to be extinguished) by blue triangles, and CS-N (not to be extinguished) by green squares.

Supplemental References

1. Sheehan D V., Lecrubier Y, Sheehan KH, Amorim P, Janavs J, Weiller E, *et al.* (1998): The Mini-International Neuropsychiatric Interview (MINI): the development and validation of a structured diagnostic psychiatric interview for DSM-IV and ICD-10. *J Clin Psychiatry* 59: 22–23.

2. Hartley CA, McKenna MC, Salman R, Holmes A, Casey BJ, Phelps EA, Glatt CE (2012): Serotonin transporter polyadenylation polymorphism modulates the retention of fear extinction memory. *Proc Natl Acad Sci U S A* 109: 5493–8.

3. Watson D, Clark LA, Tellegen A (1988): Development and validation of brief measures of positive and negative affect: the PANAS scales. *J Pers Soc Psychol* 54: 1063–1070.

4. Nelson HE (1982): *The National Adult Reading Test (NART): Test Manual.* https://doi.org/Thesis_references-Converted #319

5. Milad MR, Pitman RK, Ellis CB, Gold AL, Shin LM, Lasko NB, *et al.* (2009): Neurobiological Basis of Failure to Recall Extinction Memory in Posttraumatic Stress Disorder. *Biol Psychiatry* 66: 1075–1082.

6. Raio CM, Brignoni-Perez E, Goldman R, Phelps EA (2014): Acute stress impairs the retrieval of extinction memory in humans. *Neurobiol Learn Mem* 112: 212–221.

7. Schiller D, Kanen JW, LeDoux JE, Monfils M-H, Phelps EA (2013): Extinction during reconsolidation of threat memory diminishes prefrontal cortex involvement. *Proc Natl Acad Sci U S A* 110: 20040–5.

8. Haaker J, Golkar A, Hermans D, Lonsdorf TB (2014): A review on human reinstatement studies: an overview and methodological challenges. *Learn Mem* 21: 424–440.

9. Carleton RN, Norton MAPJ, Asmundson GJG (2007): Fearing the unknown: A short version of the Intolerance of Uncertainty Scale. *J Anxiety Disord* 21: 105–117.

10. Worbe Y, Savulich G, Voon V, Fernandez-Egea E, Robbins TW (2014): Serotonin depletion induces “waiting impulsivity” on the human four-choice serial reaction time task: cross-species translational significance. *Neuropsychopharmacology* 39: 1519–26.

11. Fernstrom JD (1979): Transport Mechanisms of Tryptophan in Blood Cells, Nerve Cells, and at the Blood-Brain Barrier. In: Baumann P, editor. *Journal of Neural Transmission, Supplement*. Vienna: Springer, pp 55–67.
